# “Stop the Bleed” Education Assessment Tool (SBEAT): Development and Validation

**DOI:** 10.7759/cureus.10567

**Published:** 2020-09-21

**Authors:** Jeffrey L Pellegrino, Nathan Charlton, Craig Goolsby

**Affiliations:** 1 Disaster Science & Emergency Services, University of Akron, Akron, USA; 2 Emergency Medicine, University of Virginia, Charlottesville, USA; 3 Military & Emergency Medicine, Uniformed Services University of the Health Sciences, Bethesda, USA; 4 National Center for Disaster Medicine and Public Health, Uniformed Services University of the Health Sciences, Bethesda, USA

**Keywords:** stop the bleed, measurement validation, rasch, tourniquet, education, competencies, first aid, disaster response and preparedness, life-threatening bleeding

## Abstract

As part of the national Stop the Bleed campaign in the United States, more than a million people have received bleeding control training through the work of many organizations. These public and professional educational experiences are ideally grounded in health sciences, clinical, and educational evidence to be most effective. However, there is currently no standard tool for evaluating the educational quality of these programs. We developed and validated the Stop the Bleed Education Assessment Tool (SBEAT) to provide a standard measure of life-threatening bleeding educational programs knowledge learning outcomes to aid in evaluation and development of this public health program.

The SBEAT development included medical, clinical, and educational experts to derive and validate learning outcomes. Specific item writing incorporated focus groups for input on language and then pilot testing before a full community pilot test established a data set, for which a Rasch methodology was applied. The resulting tool used 34 items embedded in 19 survey questions, with item separation statistic of 5.56 (0.97 reliability) and person separation statistic of 2.09 (0.81 reliability) for 171 persons. Overall, the Cronbach Alpha (KR-20) person score “test reliability” equaled 0.85 (SEM = 2.24).

The SBEAT project establishes a standardized assessment tool to evaluate the cognitive aspects of first aid for life threatening bleeding. Comparison of outcomes from different teaching styles and methods will allow for the development of best practices for future bleeding control education and help organizations demonstrate value to learners, funders, and policy makers, and advance health sciences education. SBEAT offers a measure for which educational efficiency and efficacy can be judged within a larger effort to prepare people for personal emergencies or large-scale disasters.

## Introduction

Public health, school-based, and community-based health educators work to prevent, respond, and help people recover from personal emergencies and disasters by teaching first aid. Internationally and historically, stopping life-threatening bleeding (LTB) is a core competency for first aid providers to surgeons. Although there are many causes for LTB, from road traffic accidents to acts of violence, the epidemiology of death from LTB suggests that a point-of-injury approach to stopping LTB must be improved [1-8]. In the United States the Stop the Bleed (STB) campaign launched in 2015 [9] to translate lifesaving military medical lessons learned from point-of-injury hemorrhage control to benefit the public through awareness and skill education [10-12]. After the STB campaign launch, many organizations in and around schools, as well as the community started teaching the lay public (students, faculty, staff, neighbors) and first responders hemorrhage control knowledge, skills, and behaviors.

Aligning educational outcomes and clinical evidence bases to bleeding control education took shape in 2017, when the National Center for Disaster Medicine and Public Health assembled the Stop the Bleed Education Consortium (SBEC), consisting of an international group of trauma, medical education, and public health experts. They put forward, based on analysis of existing programs, educational content and delivery recommendations for STB education programs [13], which may be applied to any LTB curriculum. The SBEC recommended three critical objectives for education programs: increase motivation of learners to act, differentiation of life-threatening from non-life-threatening bleeding, and learning to apply pressure (both direct and via a tourniquet) to stop bleeding [13]. While these recommendations have proven helpful for creating courses and vetting STB training programs, currently no validated measures exist to compare learner outcomes between or within various educational programs. Hence, it is not known which educational modalities are most effective for various learner groups, or what deficits learners may have at course completion [14-19]. In order to compare learner outcomes with objective data, and offer feedback for programmatic improvement, we developed the Stop the Bleed Educational Assessment Tool (SBEAT). The SBEAT measures the cognitive and behavioral elements needed to respond appropriately to LTB emergencies. This article describes the SBEAT’s development process and validity assessment from a Rasch approach [20] for its application in assessing any LTB education program holistically, internally and externally.

LTB competencies: a systematic approach to identify and stop hemorrhage

LTB competencies incorporate cognitive, psychomotor, and affective elements for hemorrhage control response to influence the effectiveness and willingness to help in emergencies, by moving through the Chain of Survival Behaviors [21, 22]. The challenge of assessing LTB education lies in the validity and reliability of an instrument(s) to be clinically appropriate, psychometrically sound, and educationally informative to organizations and learners [23]. The goal of LTB education, while primarily targeted to the general public, spans the population from laypeople to professionals, which adds complexity to address human factors (e.g., reading level, culture, vernacular) and clinical guideline expectations at various levels of education.

## Materials and methods

We employed a Rasch methodology for the current project to improve psychometric soundness and validate theoretical and statistical constructs measuring the cognitive elements of LTB education beyond any one curriculum [20, 24, 25]. Rasch methodology ideally is a proactive developmental approach to the instrument and ultimately examines the probability of categorical data (e.g., correct/incorrect) and models it as a function of respondent (aka person) and element being considered (aka item). The Aultman Health Foundation Human Research Review Board (2017.07 JP) approved this research project.

**Figure 1:**
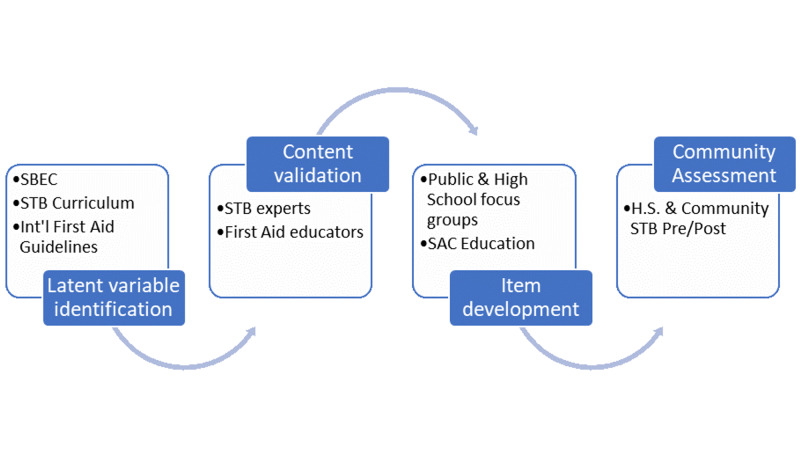
Item development strategy for SBEAT

A multi-stage approach was used to strengthen content validity of the proposed scale (see Figure 1). The first stage for assessment tool development in a Rasch approach starts with the identification of the latent variable. In this instance the latent variable consisted of the behaviors to stop LTB in a non-medical setting. The behaviors were drawn from the Stop the Bleed Educational Consortium work [13], the Stop the Bleed™ curriculum, and the international first aid guidelines [22]. In the second stage, content of a course was validated as essential by clinical and health education professionals (n = 28) through a 3-round Delphi study design, with a snowball sample. Professionals contributed to the establishment of competencies needed at various levels of training (i.e., public, lay responder, and professional) in terms of specific learning outcomes derived from the SBEC outline [13]. And they validated a variety of graphic representations (video, photos, animations). Through this phase we attempted to identify all relevant elements for assessment (see Table 1).

**Table 1:** Stop the Bleed Learning Outcomes and constructs identified through a snowball survey of experts (n = 28) LTB: Life-Threatening Bleeding; STB: Stop-the-Bleed.

	Public	Lay Responder	Professional
Individual identifies/distinguishes Life-Threatening Bleeding (LTB)	Differentiates pictures/animated bleeds representing LTB and non-LTB	Differentiates pictures/moulaged bleeds representing LTB and non-LTB	Differentiates text-based scenarios and pictures moulaged bleeds representing LTB and non-LTB
Constructs	Flow/visualized pressure of blood Volume of blood ensanguined Level of consciousness		
Individual appropriately stops the bleeding through STB competencies (i.e., with direct pressure or tourniquet, or other adjunct).	Identify LTB Initiates/Acts/Attempts to aid along the continuum of STB [Call for help/9-1-1-- Directs others or applies direct pressure on top of wound-- techniques beyond direct pressure for LTB.]	Call for help/9-1-1. Individual, Appropriately able to stop the bleeding through STB competencies (i.e., firm, steady direct pressure or tourniquet- commercial/ improvised, or other adjunct).	Demonstrate appropriate hemostasis skills on core, head, and extremities: - direct pressure (manual & mechanical); - accessible tourniquets; - packing/ hemostatic agents.
Constructs	Location amenable to tourniquet placement, placement locations for tourniquet, awareness to apply pressure/ tighten tourniquet till hemostasis, sequence apply pressure/tourniquet, applying tourniquet through pain caused, problem solving if original tourniquet placement does not work or loosens		

In the third stage, community participants (n = 187) contributed to item development. The artifacts used throughout the study, such as the visuals, words, metaphors, and sample questions, were taken to communities of interest in focus groups. These focus groups allowed us to strengthen the tool by clarifying meanings of words, and gain knowledge on plain or vernacular language that would help better represent medical terms and constructs. For example, hemorrhage control experts identified 6 oz (≈177 ml) of blood loss to represent LTB for lay responders. While this particular amount of blood loss may not be inherently life-threatening for most individuals, the presence of this volume of blood in the setting of continued bleeding could lead to rapid clinical deterioration. This is different than a blood donation, for example, in which the bleeding is controlled and will stop. In an injured person, this volume represents life-threatening bleeding that might benefit from rapid layperson intervention.

People while looking at a 6 oz pool of “blood” described it in three different categories. Volume was described in various measurements including ounces, gallons, and liters. Graphically people described it in terms like “a lot”, big, or puddle. And, in emotional terms that included “crap load”, “oh damn.” This variety impressed upon us the need to have multiple representations to describe LTB when asking people to identify it. During a citizen-science-project with high school students and faculty (n = 142) at a conference, individuals polled model items and electronically provided feedback on illustrations (word-clouds, open text) before group debriefing. Feedback provided an opportunity to revise the question format and word choices. For example, a picture of a commercial tourniquet on a non-bleeding limb was mis-identified for something other than a hemorrhage control device by more than 60% of the 142.

From this preparation, we developed a series of items for the cognitive and competency domains that were presented for peer review to the Education and First Aid sub-councils of the national Scientific Advisory Council (SAC) of the American Red Cross (n = 18). Then items were pilot tested (n = 77) to discern final language and domain appropriateness, to build construct validity.

**Figure 2:**
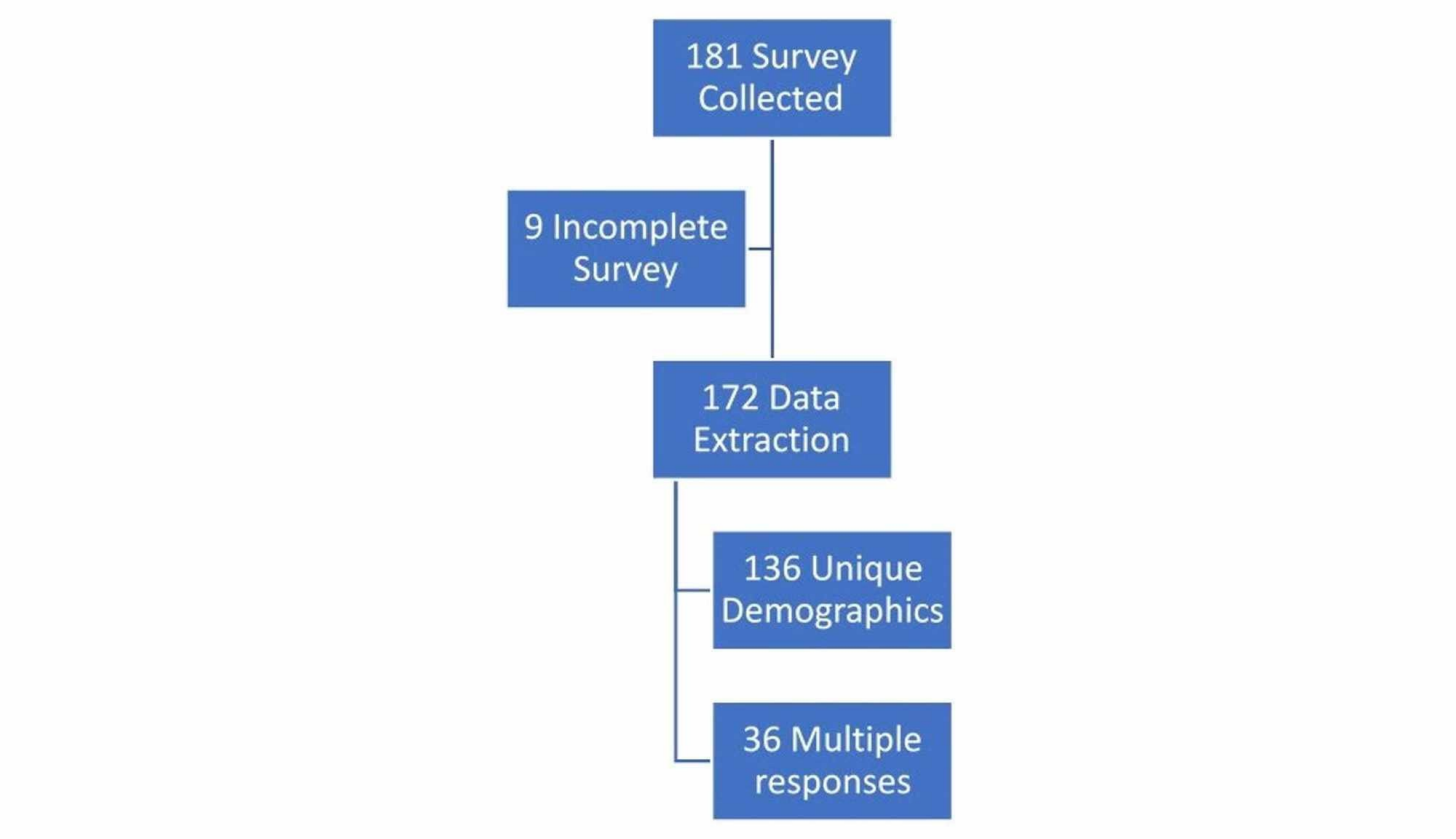
Community pilot data collection

This report summarizes stage four, the Community Assessment for validation. Data was collected September-October 2019 from 16 STB courses. Courses consisted of 1-2 trainers, both with 20+ years of community and professional education experience, lasting no more than 60 minutes, covering the learning outcomes of Stop the Bleed®. Participants took pre-learning assessment 3-7 days prior to face course. Two courses had STB built into a full day first aid course. In total, we extracted data from 172 surveys for analysis (see Figure 2).

Analysis for reliability and validity

We utilized Qualtrics software (Provo, UT, USA), version October 2019, to collect data, with data transformed into binary responses in Microsoft Excel and then uploaded into Winsteps (4.4.7) for analysis. Rasch scaling and modeling uses a mathematical formula to describe the relationship between persons (participants) and items (survey responses) to limit the bias of samples and provide a true interval scale estimation [20]. B_n_-D_i_ = ln(P_ni_/1-P_ni_) articulates the Rasch mathematical model for dichotomous test items, “based on an appreciation that, to make measurements in the case of right/wrong test items, researchers must consider the difficulty of each test item along a variable and the overall ability level of a test taker with respect to the variable” [25]. Therefore, when a potential first aider (B_n_) answers an item (D_i_), this relationship will be expressed by the natural log of the respondent correctly answering the item (P_ni_) divided by the probability of the respondent not correctly answering the test item (1 − P_ni_).

Fit statistics of persons and items provide an indication to the appropriateness (quality control) of the overall tool and each person and item by showing the size of randomness or distortion. The Mean-square expected value (MNSQ) is 1.0, with values <1.0 identifying persons or items that are too predictable and >1.0 being unpredictable [26]. “Infit means are less sensitive than outfit means to extreme responses” [27]. The Means Square Index with values between .50 and 1.50 are acceptable and more preferred as they approach 1.0. If items depart from these parameters, then it alerted us to responses to items that may not be indicative of the STB constructs (e.g., indicative of perhaps reading level or experience).

Next, overall person and item “separation and reliability” provided a perspective of the variable on a continuum. Person separation <2 and reliability <0.8 implies that a survey may need more items because the instrument may not be sensitive enough to distinguish all participants. The advantage of significant person separation >2 is being able to analyze participant response patterns independently of the reliability of the survey items. Item separation <3 and reliability <0.9 implies that the participant sample is not large enough, which limits construct validity [28]. The Cronbach Alpha (KR-20) traditionally is reported as the conventional “test” reliability index in Rasch methodology.

**Figure 3:**
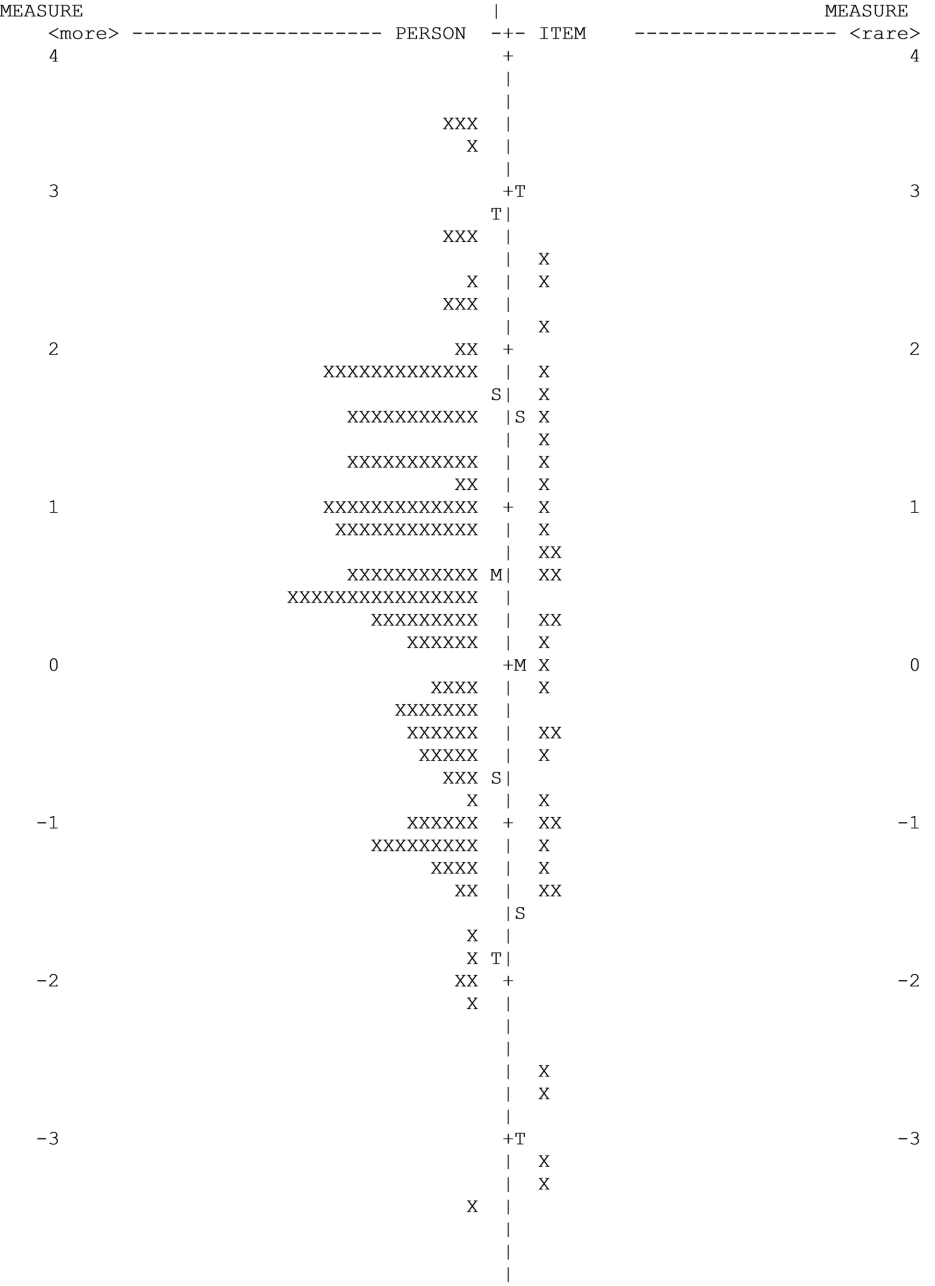
SBEAT Wright map of 171 persons and 34 items Person = participants, Items = dichotomous response. M = Mean; S = 1 Standard Deviation; T = 2 Standard Deviations Created in WINSTEPS 4.4.4

Finally, detecting and evaluating the impact of multidimensionality uses item fit statistics and principal component analysis of residuals (PCA) (www.winsteps.com/winman/principalcomponents.htm). From a Rasch development model, the goal is to limit contrast (number of variables) and to focus on the variable of interest. This process adds to the validity and reliability for the items measuring the same variable or understanding that there may be alternative variables that need to be explored and segregated. Once items demonstrate unidimensionality and free from bias, the Rasch model places all items on a hierarchical linear scale, with the most simplistic items, the lower end, to the most difficult STB underlying concepts, at the upper end. This is plotted against participants ranging from least knowledgeable, lower end, to the most knowledgeable, at the upper end (see Figure 3).

After data are transformed through Rasch modeling to a linear measure, yielding individual Person Measures (Winsteps, 2020) they provide person ability estimates that may be statistically assessed for scale measurements (e.g., t-test). Scores can range from -4 to 4, where 0 refers to a 30% probability of success. Independent t-tests were then performed in IBM SPSS software version 26.0 (IBM Corp., Armonk, NY) to identify differences between groups in pre- and posttest scores. Cronbach’s alphas were determined to identify difference between groups and categorical results with Pearson measures reported. We hypothesized that sample characteristics of gender, race, educational level would not have statistical differences between the standardized Rasch Person Measures. We further hypothesized that level of first aid training and specifically hands on training with a tourniquet would be different than those without such training.

## Results

Using a Rasch approach, pre and post course SBEAT administrations signify individual responses because the person should be different based on any intervention. In this Community Pilot, 181 surveys were submitted, nine were substantially incomplete, leaving 172 for analysis. Demographics were collected from 136 participants (see Table 2), representing individuals taking the instrument the first time. Recruitment happened predominantly with high school and college students resulting in a sample that was younger, Caucasian, and female with a diversity of previous first aid training, including tourniquet training with practice (11%).

**Table 2:** Demographic characteristics of SBEAT respondents (n = 136)

Characteristic	Frequency (n)	Percentage
Female	79	58.1
Male	52	38.8
Caucasian	110	80.2
No College Degree	121	89.0
First Aid Trained	62	45.6
Tourniquet Trained	36	26.5
Age	mean 20.6 years (SD 12)	

The 83 individual items’ Infit measures (MNSQ) fell within the acceptable ranges (person 0.99 and item 1.00). The original modeled data reported person separation statistic of 2.27 (0.84 reliability) for 171 persons (one person dropped from analyses because of a perfect score) and item separation statistic of 5.10 (0.96 reliability) for all 83 items. In examining the Disattenuated (Pearson) Correlation, with extreme scores removed, showed about half as much variance as they did independently, meaning possible different variables between the three clusters identified as well as a negative correlation between clusters, however the unexplained variance all fell below three eigenvalues (PCA), representing that these were a combination of random and accidental variance.

Of the 83 items, several were redundant in differentiating individuals, as well as redundant in measuring a learning outcome. Refinement of the model commenced to reduce the number of items to a meaningful measure that would be practical to administer in the field. The final model for SBEAT utilized 34 items found within 19 survey questions (see Appendix A). In this version, all the items fell below the 1.5 threshold for fit statistics; however, five items fell below the 0.5 MNSQ, with the average Outfit of 0.85 for both person and item. Upon investigation, the five items all did not change the overall variance explained by measures (34%), as well as provided a lower anchor for the scale to improve person separation, and thus were kept. All the Infit measures (MNSQ) fell within the acceptable ranges (person and item 1.00).

The SBEAT modeled data reported a person separation statistic of 2.09 (0.81 reliability) for 171 persons (one person dropped from analyses because of a perfect score) and item separation statistic of 5.56 (0.97 reliability) for the 34 items. Overall, the Cronbach Alpha (KR-20) person score “test reliability” equaled 0.85 (SEM = 2.24). Additionally, using Microsoft Word, readability statistics computed to be easily understood by 13-15-year-old students (Flesch Reading Ease 72.1; Flesh-Kincaid Grade level 6.3).

Relationship to other variables

Design of the SBEAT follows the two expectations of Rasch modeling calibration and measurement: “(1) that a more able person should always have a greater probability of success on any item than a less able person, and (2) that any person should always be more likely to do better on an easier item than on a harder one” [29]. In the SBEAT Wright map, persons are represented in a hierarchy of low to high performers. Items, similarly, are presented from easy to hard (see Figure 3). A person score adjacent to an item score correlates to that a person having a 30% chance of answering the item correctly. We chose this metric over the default 50% chance because SBEAT users need greater assurance; it is the competency and not chance being measured.

The average person score (n = 171) was 0.51. Gender, race, and educational level did not differ significantly in Person Measure. Groups we hypothesized being different are summarized in Tables 3-5.

**Table 3:** Person measure between participants with no first aid training and first aid trained STD = Standard Deviation

	No Previous First Aid Training (n = 75) (STD)	First Aid Trained (n = 60) (STD)	
Mean Rasch Person Measure (Std Deviation)	0.170 (1.15)	0.535 (1.26)	p = 0.081

**Table 4:** Person measure between participants trained in tourniquet use with and without practice * = statistical significant < p = 0.05 STD = Standard Deviation

	Tourniquet Trained - No practice (n = 17) (STD)	Tourniquet Trained - With practice (n = 19) (STD)	
Mean Rasch Person Measure (Std Deviation)	-0.1365 (1.38)	0.95 (1.53)	p = 0.032*

**Table 5:** Person measure difference between participants with prior Stop the Bleed (STB) training and those immediately post education *= Statistical significant < p = 0.05 STD = Standard Deviation

	Prior to STB Training (n = 42) (STD)	Just Completed STB Training (n = 48) (STD)	
Mean Rasch Person Measure (Std Deviation)	0.373 (1.16)	0.880 (0.99)	P = .0280*

## Discussion

We explored simple to complex cognitive and behavioral survey items and their variations to represent the competencies for treating LTB. The rigors of soliciting expert clinicians and educators in identifying learning outcomes reduced the chance of including non-relevant or distracting elements, thus increasing content validity. The inclusion of community input into the structure of building items helped build stronger construct validity arguments by appropriating non-medical language that is accessible to people with post-elementary education. Our substantive theory follows a pattern of being able to recognize an LTB from environmental context (what do you see) and the victim (how are they acting), then choosing an appropriate action based on the injury (use of pressure or tourniquet), and finally the sequence of applying pressure or a tourniquet, including actions in case hemostasis is not achieved. These sequenced competencies also represent modules within various LTB curricula.

The initial Community Pilot of 83 items calculated a good overall Cronbach alpha (KR-20) for person raw score of 0.88 (SEM = 3.33). However, administering the pilot to solicit all 83 items took an inordinate amount of time within an hour-based curriculum. We thus refined the original model into what is now the SBEAT by eliminating variations of the same concept, resulting in 34 items from 19 questions in the tool, representing each of the original categories. In the end, person separation achieved 2.09 with a Cronbach alpha of 0.81 (SEM = 2.24) reliability indicating the internal consistency of the scale. Similarly, we worked to have an appropriate Item separation to verify item hierarchy. The high item separation (5.56) in the model implies a large enough person sample to confirm item difficulty hierarchy/construct validity of SBEAT [28]. Unidimensionality was confirmed as the eigenvalue plot (i.e., scree plot) showed one dominant factor and explained at least 34% of the variance and the contrasts (factors) loadings were under three eigenvalues.

The sample, although young, represented a large audience for STB education in schools and community. The diversity of first aid experience demographically, and specifically with tourniquets contributed to assessing our hypotheses for discrimination by the tool, when compared to demographics that should not influence STB competencies (gender, race, or educational level). Relationship to other variables is an important element when developing a novel tool because other tools are not comparable. With our hypothesis testing we established a high sense of connection as those with more experience in first aid education and specifically tourniquet training performed higher on the SBEAT. This then was followed by people with practice with tourniquets measure significantly higher than those only introduced to tourniquets. Although there is not a discrete or perfect correlation or exclusiveness to the level of training, this in vivo sample represents a range of learners that exist as potential or actual participants. The final SBEAT, as a whole, offers administrators of the tool content and construct validity for use in assessing knowledge and intended behaviors for LTB irrespective of social demographics [30].

SBEAT application

The SBEC aims to standardize the features of bleeding control programs in order to maximize participant success and increase public safety. SBEC recommended establishing tiered training categories for education domains, objectives, definitions, content, educational design, and assessment to motivate learners to act when faced with a hemorrhage emergency, distinguish life-threatening hemorrhage, and learn to apply pressure. The SBEC also suggested educational design including web-based, in-person didactics and in-person skill for different levels of training. SBEAT can be used to comparatively evaluate the learning outcomes of each of these educational designs/modalities, as well as evaluating between tiers of training for all levels of learners.

Rasch measures are reported differently than percentages of correct vs. incorrect responses, which on the surface maybe confusing to those trying to understand the output. Person measures range from -4 to 4, with zero being the mean, and correlate to the item measure. The Wright map provides a picture of the exam items and ability of the SBEAT participants on an interval scale. Theoretically, when participants and items are opposite each other on the map, the difficulty of the item and the ability of the candidate are comparable, so the candidate has approximately a 30% probability of answering the item correctly. More important to the application of SBEAT is the Person Measure to linear statistical analysis.

Psychometrically, we can identify varying amounts of STB competencies in individuals and consequently in educational interventions, with appropriate samples, using the SBEAT. As educational and public health organizations utilize SBEAT, part of the value may be in the development of pretest/posttest versions for completion or certification. The use of Rasch methodology encourages future item development, for which pretest/posttest equivalency can be assessed; assuming each measure remains part of a single variable because it is on a single scale [20]. The development of new items may also serve to keep the instrument fresh and reduces the chance that organizations only teach to specific test items versus larger concepts. All new items should be piloted and then plotted using Rasch analysis.

Moving forward, SBEAT should be centralized for free web-based administration to organizations that can use it to assess and improve their learners’ outcomes based on LTB competencies and local implementation. Additionally, the use of outcomes from a common tool allows assessment between training programs, from which guidelines can be developed. From this effort standard setting activities may also establish mastery levels outcomes at various training levels.

Limitations

As with most first aid educational assessments, SBEAT validity did not include observation of participants in LTB situations. Nor were the individual skills of learners assessed before or during STB education evaluated in relationship to SBEAT measures. We utilized the expertise of individuals with actual experience and practice of STB competencies to establish construct validity of SBEAT. There is also a purposeful lack of categorical definition for educational success described by this instrument, as any threshold value of a certification or documentation of completion of a STB course needs to be valued by an organization in context of the learner and expectations for results. For example, legally obligated responders may need a higher level of performance, where a community member might be judged successful if they know when to call for help, while applying pressure.

The tool may also be limited at the more knowledgeable end as people may obtain a perfect score. Based on the development process we are professionally comfortable with people demonstrating up to the maximum score as there is a finite knowledge of bleeding and control competencies in the first aid domain. The Person and Item separation scores however discriminate within and between groups at various levels of training, which add to the overall value of the tool. However, additional tools are needed to measure the intention to help and skill assessment components to achieve a comprehensive educational assessment of a curriculum, with the SBEAT being a primary component.

In some cases of first aid education, assessments provide a level of feedback to the learner to encourage a person to learn more or feel confident about their level of knowledge, however this development and validity study did not have this intent. If educational organizations desired to do this, modifications to scoring and application of SBEAT would need to take place. We chose to apply a Rasch Analysis to the data, which we believe are equally valued and reliable and not subject to interference from extraneous variables, however other forms of assessment need to now take place in terms of applying it to actual learning outcomes, including intention to aid, to holistically assess all SBEC outcome recommendations.

## Conclusions

We have developed and validated the first universal assessment tool for knowledge and competencies to stop LTB. The SBEAT project establishes a standardized assessment tool to evaluate the cognitive aspects of the SBEC objectives and universal first aid competencies. The SBEAT also models a Rasch approach for validating a competency in education. Additional work is needed to merge the motivational and psychomotor aspects of STB competencies. We believe SBEAT to be validated and implementable across training programs to determine the level of learning outcomes of individual students for program assessment and between programs. Comparison of different teaching styles and methods will allow for the development of best practices for future bleeding control education and help organizations demonstrate value to learners, funders, and policy makers. In addition, with a validated widely disseminated assessment, curriculum designers can gather the information needed to continue to design and advance training in bleeding control.

## Appendices

Appendix A: Stop the bleed educational assessment tool (SBEAT) items

Disclaimer

The items below are meant to be transparent in the design of the tool. It is not a comprehensive assessment of knowledge, skills, or attitude. And it is not validated to be scored as a summary or total percentage scored. The responses need to be subjected to a Rasch analysis, from which person scores may be derived for secondary assessment, per the original article to separate individuals. The authors are willing to share and/or collaborate on future use of the tool by researchers or training organizations.

[To the participant ] “Life-threatening bleeding” (LTB) is an emergency. It is an injury or wound that should be cared for first because the loss of blood is great enough that the person will die if no first aid is given or delayed.

Keep this in mind as you answer the following questions.

This first section is about being able to identify LTB (Life Threatening Bleeding) from a variety of descriptions:

Which of the following need immediate first aid to stop the bleeding:

Options:

Illustration of Blood oozing from scraped knee

Illustration of Blood spurting from arm

Illustration of Blood pooling on ground

Illustration of Blood gushing from amputated foot

Which of the following does not need immediate first aid to stop the bleeding:

Options:

Spurting/pulsating blood from the injury

Streaming blood from the injury

Oozing/trickling blood from injury

Gushing blood from the injury

Which of the following need immediate first aid to stop the bleeding:

Options:

Photo of Scrape from a fall

Photo of full thickness burn

Photo of Cut to thigh

GIF file of Spurting blood from leg

Which of the following need immediate first aid to stop the bleeding:

Options:

Blood spotting through one piece of gauze

Blood soaked clothing or bandages

Bleeding that is not soaking through a bandage

The overlying clothes are soaked in blood

Which of the following need immediate first aid to stop the bleeding:

Options:

Pooling of blood on the ground

Enough blood to fill 1/2 a soda can

Two-tablespoons of blood from the injury on the ground

A hole in the skin without flowing blood

Which of the following need immediate first aid to stop the bleeding:

Options:

Dripping blood from a cut

Gushing blood that will not stop coming out, even with a hand pushing on it

Flowing blood from an injury that you are pressing on

Blood spotting through several layers of cloth

Rank the following: 1= at the top, most likely to die-- 4= at the bottom least likely to die (slide options)

Options:

Bleeding from a person who is now unconscious

Bleeding from a person who is not alert/not fully awake anymore

Bleeding from a person who is now confused/not acting normally

Bleeding from a person who is now pale

[*Explainer to participant*] A tourniquet is a device that, when properly applied, stops blood from flowing by compressing blood vessels to a bone. [representative photos of a commercial Combat Application Tourniquet and an improvised tourniquet, both placed on a forearm]

A tourniquet can be applied to a LTB in which of the following areas?

Options:

Leg

Arm

Hand

Stomach

Which of the following need a tourniquet applied to save the life:

Options:

Photo of Scrape from a fall

Photo of full thickness burn

Photo of Cut to thigh

GIF file of Spurting blood from leg

What is the best means to stop life-threatening bleeding on a leg?

Options:

Tell the injured person to apply pressure to the wound while you go look for a tourniquet

Apply an improvised tourniquet using a roll of twine

Stuff the wound with a tampon

Wrap the leg and over the wound with packing or duct tape

A tourniquet may be required for an arm/leg that is partially or fully torn off.

Options:

True

False

[*To the participant*] This next section is about the actions of using pressure and tourniquets to stop LTB.

Climbing over a fence, a person cuts their stomach; it is bleeding and 3-4 inches long. To control bleeding from this wound, you should:

Options:

Apply firm pressure with your hand directly where the bleeding seems to coming from

Apply a pressure dressing first, then apply manual pressure over the pressure dressing

Apply an elastic bandage around the abdomen, ensuring that the wound is covered

Apply a cold pack to the wound

A person falls and is now bleeding on the back of their head. To control the bleeding, you should:

Options:

Apply pressure with one finger tip

Apply multiple layers of gauze to the wound and secure with a roll of gauze

Apply a warm pack/compress

Use a pressure point such as the temporal artery to control bleeding

If you are alone with somebody who is not awake and has LTB coming from an arm/leg, what best describes your first action(s)?

Options:

Call for help on a cell phone

Put a tourniquet on the injury

Call for help while apply pressure or placing a tourniquet (i.e., speaker phone, yelling, activating alarm)

Put the tourniquet above the wound so that it is between the torso/body and the wound.

Options:

True

False

After placing the windlass tourniquet above the injury, it is critically important to tighten it as much as possible before turning the stick/rod.

Options:

True

False

You have applied a tourniquet to a LTB on a leg. The bleeding appeared to stop initially, but has now restarted. Which of the following should be the next step:

Options:

Apply a second tourniquet above the first (between the first tourniquet and the torso)

Check to see if the tourniquet has loosened and tighten the tourniquet by turning the windlass/rod more

Loosen the tourniquet, then reapply closer to the wound

Remove the tourniquet completely and apply a different type of tourniquet

Apply a second tourniquet directly on top of the first tourniquet

If the person is in pain, the tourniquet is on too tight.

Options:

True

False

When should a tourniquet be removed?

Options:

A tourniquet should never be removed unless instructed by a physician or EMS provider

When the patient begins experiencing severe pain

When a first responder arrives on scene

When bleeding has stopped

When there no pulse in the hand or foot

When packing a bleeding injury, it is important to push material as close to the bleeding vessel as possible.

Options:

True

False

After packing an injury to stop bleeding, it is not necessary to apply pressure any more.

Options:

True

False
